# The acetyltransferase SCO0988 controls positively specialized metabolism and morphological differentiation in the model strains *Streptomyces coelicolor* and *Streptomyces lividans*

**DOI:** 10.3389/fmicb.2024.1366336

**Published:** 2024-07-24

**Authors:** Yunwen Bi, Hao An, Zhewei Chi, Zhongheng Xu, Yuan Deng, Yuxian Ren, Rui Wang, Xinyi Lu, Jia Guo, Ren Hu, Marie-Joelle Virolle, Delin Xu

**Affiliations:** ^1^Department of Ecology, Institute of Hydrobiology, School of Life Science and Technology, Key Laboratory of Eutrophication and Red Tide Prevention of Guangdong Higher Education Institutes, Engineering Research Center of Tropical and Subtropical Aquatic Ecological Engineering, Ministry of Education, Jinan University, Guangzhou, China; ^2^Université Paris-Saclay, CNRS, CEA, Institute for Integrative Biology of the Cell (I2BC), Department of Microbiology, Group “Energetic Metabolism of Streptomyces”, Gif-sur-Yvette, France

**Keywords:** acetyltransferase, SCO0988, *Streptomyces*, acetylome, BldKB

## Abstract

Streptomycetes are well-known antibiotic producers possessing in their genomes numerous silent biosynthetic pathways that might direct the biosynthesis of novel bio-active specialized metabolites. It is thus of great interest to find ways to enhance the expression of these pathways to discover most needed novel antibiotics. In this study, we demonstrated that the over-expression of acetyltransferase SCO0988 up-regulated the production of specialized metabolites and accelerated sporulation of the weak antibiotic producer, *Streptomyces lividans* and that the deletion of this gene had opposite effects in the strong antibiotic producer, *Streptomyces coelicolor*. The comparative analysis of the acetylome of a *S. lividans* strain over-expressing *sco0988* with that of the original strain revealed that SCO0988 acetylates a broad range of proteins of various pathways including BldKB/SCO5113, the extracellular solute-binding protein of an ABC-transporter involved in the up-take of a signal oligopeptide of the quorum sensing pathway. The up-take of this oligopeptide triggers the “bald cascade” that regulates positively specialized metabolism, aerial mycelium formation and sporulation in *S. coelicolor*. Interestingly, BldKB/SCO5113 was over-acetylated on four Lysine residues, including Lys^425^, upon SCO0988 over-expression. The bald phenotype of a *bldKB* mutant could be complemented by native *bldKB* but not by variant of *bldKB* in which the Lys^425^ was replaced by arginine, an amino acid that could not be acetylated or by glutamine, an amino acid that is expected to mimic acetylated lysine. Our study demonstrated that Lys^425^ was a critical residue for BldKB function but was inconclusive concerning the impact of acetylation of Lys^425^ on BldKB function.

## 1 Introduction

*Streptomyces* are widely distributed in terrestrial, marine and freshwater environments where they play important ecological roles (Chater, 2016). *Streptomyces* undergoes a complex morphological differentiation cycle starting with the germination of a spore that yields a substrate mycelium from which aerial hyphae arise. Subsequently, the tip ends of these aerial hyphae differentiate into spores. This morphological differentiation process is accompanied by the production of a variety of specialized bioactive metabolites of great interest in modern medicine (antibacterial, anticancer, and immuno-suppressive drugs) or agriculture (fungicides, herbicides, pesticides etc.…) (Niu et al., 2016). Indeed, more than 60% of clinically used antibiotics are original or chemically modified versions of metabolites produced by *Streptomyces* species (Lewis, 2020). A given *Streptomyces* specie is usually known to produce less than 5 characterized bio-active metabolites whereas the *in silico* analysis of the sequenced genome of numerous *Streptomyces* species revealed the presence of 5 to 10 fold more biosynthetic pathways that are likely to direct the biosynthesis of yet unknown specialized metabolites (Ikeda et al., 2014; Hannigan et al., 2019). Most of these pathways are unfortunately not expressed (cryptic) under normal laboratory conditions. In consequence, only a small fraction of the potential biosynthetic capacity of these bacteria is known and thus exploited. The implementation of novel strategies to activate the expression of these silent pathways is necessary to exploit the huge metabolic diversity of this genera and discover most needed novel antibiotics to face the worrying emergence and rapid spreading of antibiotic-resistant pathogens.

Several strategies were used to increase the expression of specialized metabolites biosynthetic pathways. These include genetic manipulations of genes encoding regulators linked or not to the pathways, use of elicitors or co-cultivation with other microorganisms (Ochi, 2017). However only few studies were conducted to determine the impact of post-translational modifications (PTM) on the function of proteins playing a role in the regulation of primary and specialized metabolism in *Streptomyces* species (Zhang and Xu, 2018). That is only in the last decade that extensive post-translational proteins modifications were discovered in *S. coelicolor* and in other *Streptomyces* species (Hesketh et al., 2002). These modifications include the phosphorylation of His and Asp residues of sensory kinases and response regulators of two component systems (Cruz-Bautista et al., 2023) or that of Ser or Thr residues of various proteins by eukaryotic-like Ser/Thr kinases (Wright and Ulijasz, 2014) but these modifications can also include succinylation (Yang et al., 2021), crotonylation (Sun et al., 2020b) as well as acetylation (Liao et al., 2014; Hamam et al., 2019).

*Streptomyces coelicolor* genome encodes 93 acetyltransferases (Bentley et al., 2002) but the biological function of most of them is unknown. Indeed, only a few studies demonstrated the involvement of acetyltransferases in the regulation of enzymes activities, protein–protein or DNA-transcriptional factors interactions in *Streptomyces* (Martín and Liras, 2020). In one of our previous published studies, we identified the acetyltransferase SCO0988 as a regulatory target of SCO3201, a regulator of the TetR-family whose over-expression caused a strong inhibition of the biosynthesis the blue polyketide antibiotic actinorhodin (ACT) and of sporulation in *S. coelicolor* (Zhang et al., 2020). Since the expression of *sco0988* was repressed in condition of *sco3201* over-expression (Xu et al., 2010), this suggested that SCO0988 might have a positive impact on ACT production and sporulation. In order to test this hypothesis, *sco0988* was deleted in *S. coelicolor* (*SC*), and over-expressed in *S. lividans* (*SL*). *SC* and *SL* are phylogenetically closely related model strains that bear the same pathways directing the biosynthesis of the colored antibiotics, undecylprodigiosin (RED), a red-pigmented tripyrrole antibiotic (Feitelson et al., 1985), and actinorhodin (ACT), a blue pigmented polyketide antibiotic (Malpartida and Hopwood, 1986). However, these metabolites are abundantly and poorly produced by *SC* and *SL*, respectively. We demonstrated that the deletion of *sco0988* in *SC* abolished antibiotic production and sporulation of *SC* whereas its over-expression in *SL* enhanced the weak antibiotic production of this strain as well as its sporulation. Our study thus confirmed that SCO0988 has a positive impact on both metabolic and morphological differentiation of these two model species. A comparative analysis of the acetylome of *SL* over-expressing SCO0988 with that of the native strain revealed the acetylation of lysine residues present in proteins belonging to diverse ontological classes. We decided to focus our study on BldKB/SCO5113, an extracellular solute-binding protein of an oligopeptide ABC transporter for two main reasons. Firstly, the intensity of acetylation of this protein was greatly enhanced upon SCO0988 over-expression and secondly because the BldK transporter is known to be involved in the bald signaling cascade of the quorum sensing pathway that plays a positive role in the control of antibiotic production and morphological differentiation in *SC* (Nodwell et al., 1996). Our results suggested that the acetylation of BldkB on the Lys 425 residue is necessary for its functionality and the way this acetylation could impact BldKB function is discussed. Our study thus contributes to a better understanding of the role of the acetyltransferase SCO0988 in the activation of antibiotic production and morphological differentiation in *Streptomyces* species.

## 2 Materials and methods

### 2.1 Bacterial strains and culture conditions

The strains used in this study were *S. coelicolor* M145 and *S. lividans* TK24 (Bentley et al., 2002). These strains were grown at 28°C in MS (ACROS, Belgium) and YEME media (ACMEC, Shanghai) for spore generation and protoplast preparation, respectively, whereas R2YE medium (BD, United States) was used for protoplasts regeneration after transformation (Kieser et al., 2000). Apramycin, and thiostrepton (HARVEYBIO, Beijing) were added in solid R2YE medium at a final concentration of 50 μg/mL in cultures of *SC* or *SL* containing the plasmids pOJ260 and pSET152 and the plasmids pDH5 and pWHM3, respectively. The following *E. coli* strains were used, DH5α for routine cloning, ET12567 to prepare de-methylated plasmids to transform *SC* and C43 for efficient protein expression. These strains were grown in Luria broth (LB) medium. Ampicillin (50 μg/mL), chloramphenicol (25 μg/mL), and kanamycin (50 μg/mL) were added to growth media when required.

### 2.2 Construction of a *Streptomyces coelicolor* strain deleted for *sco0988* encoding an acetyltransferase

In order to disrupt the gene encoding the acetyltransferase SCO0988 in *S. coelicolor* M145 (*SC*), a 370 bp DNA fragment internal to the *sco0988* coding region was amplified by PCR using the primer pair DisC*sco0988*-F/DisC*sco0988*-R (Table 1) and *SC* chromosomal DNA as template. The resulting PCR fragments were digested by *Bam*HI and *Hind*III (Takara, Japan) and ligated into the plasmid pOJ260 that carries a gene conferring resistance to apramycin cut by the same enzymes (Kieser et al., 2000). The resulting plasmid, pOJ260-*sco0988^int^*, was transformed into *E. coli* ET12567 to prevent its methylation (Flett et al., 1997) and apramycin resistant (Apra^R^) transformants were selected. The non-methylated plasmids extracted from the Apra^R^ transformants were transformed into *SC* and selected for Apra^R^. The *sco0988* disrupted mutants (*SC*-∆*sco0988*) were confirmed by PCR using the primer pair DisC*sco0988*-F/DisC*sco0988*-R (Table 1).

**Table 1:** Synthetic oligonucleotides used in this study.

Primer	5′ → 3′ sequence	Description
DisC*sco0988*-F	CGGGATCCTGGAACGAGGACGAGGAGAA	Amplification of the *sco0988* coding sequence for disruptation in *S. coelicolor*
DisC*sco0988*-R	CCCAAGCTTGGTGGTAGAAGGCCATCGC	
OE*sco0988*-*Xba*I-F	GCTCTAGACTGCTGGGCCTGATCC	Amplification of the *sco0988* coding sequence for overexpressing in *S. lividans*
OE*sco0988*-*Hind*III-R	CCCAAGCTTCCGTAGGGCTTCCTGAGT	
DelUp*sco5113*-*Hind*III-F	CCCAAGCTTGCGTGCTCCTCTTTGC	Amplification of the *sco5113* coding sequence for In-frame deletion in *S. coelicolor*
DelUp*sco5113*-*Xba*I-R	GCTCTAGACGACTGCGACTTGGCGT	
DelDown*sco5113*-*Xba*I-F	GCTCTAGATACATCTCCAAGGAAGTGAA	
DelDown*sco5113*-*Kpn*I-R	CGGGGTACCCTTCGGGCTTGGTCTGAGT	
VerifDel*sco5113*-F	GAGCATTCTCCGTAACCG	Verification of the *sco5113* mutant which used the way of In-frame deletion in *S. coelicolor*
VerifDel*sco5113*-R	CGTGTCCCAGACGTTCT	
CP*sco5113*-*Eco*RI-F	CGGAATTCAGGGAAACCGCTGAGG	Amplification of the complementation of *sco5113* (AAG)*/sco5113* (AGG)/sco5113 (CAG) in *SC-*Δ*bldKB* and the construction of pUC19-*bldKB* (AAG) & pUC19-*bldKB* (AGG) & pUC19-*bldKB* (CAG)
CP*sco5113*-*Xba*I-R	GCTCTAGAGCCACCGTTTCCCGA	
K425R*sco5113*-F	AGGCCGCCAAGAGGCTCATCAAGGAAGGCGGCT	Amplification of the acetylation site mutation in pUC19-*bldKB* (AAG)
K425*Rsco5113*-R	TCCTTGATGAGCCTCTTGGCGGCCTCGATGTCG	
K425Q*sco5113*-F	AGGCCGCCCAGAAGCTCATCAAGGAAGGCGGCT	Amplification of the acetylation site mutation in pUC19-*bldKB* (CAG)
K425Q*sco5113*-R	TCCTTGATGAGCTTCTGGGCGGCCTCGATGTCG	
PE*sco5113-Eco*RI-F	CGGAATTCGCTCACTACTCACAGGCAG	Protein expression of SCO5113 using pET28a
PE*sco5113-Hind*III-R	CCCAAGGTTCTTCTTGAGGAAGACCCG	

### 2.3 Construction of the plasmid to over-express *sco0988* in *Streptomyces coelicolor* M145-∆*sco0988* and in *Streptomyces lividans* TK24

In order to complement the *SC*-∆*SCO0988* mutant and to over-express *sco0988* in *S. lividans* TK24, the coding sequence of *sco0988* was amplified by PCR using the primers OE*sco0988*-*Xba*I-F and OE*sco0988*-*Hind*III-R (Table 1) and *SL* genomic DNA as template. The resulting PCR products were digested by *Xba*I and *Hind*III and cloned into pWHM3-*ermE* cut by the same enzymes in order to put the expression of *sco0988* under the control of the strong *erm*E promoter (Bibb et al., 1985). The resulting pWHM3-*ermE*-*sco0988* plasmid was transformed in *SL* and in *SC*-∆*sco0988* and protoplasts to generate the strains *SL*/pWHM3-*ermE*-*sco0988* and *SC*-∆*sco0988*/pWHM3-*ermE*-*sco0988*. The *SC* and *SL* strains harboring pWHM3-*ermE* empty plasmid were used as a control (see Table 2).

**Table 2:** Plasmids and strains used in this study.

Plasmid and strain	Description	Reference or source
**Plasmids**		
pOJ260	*E. coli* plasmids without *Streptomyces* replicon or integration function; Apra^r^	Kieser et al. (2000)
pOJ260-*sco0988^int^*	pOJ260 carrying the *sco0988* (217–586) genes	This study
pWHM3-*erm*E	High-copy-no. *E. coli-Streptomyces* shuttle expression vector carrying the *erm*E promoter; Tsr^r^, Amp^r^	Bibb et al. (1985)
pWHM3-*erm*E-*sco0988*	pWHM3-*erm*E carrying the *sco0988* gene cloned downstream of the *erm*E promoter	This study
pDH5	High-copy plasmid; Tsr^r^	Kieser et al. (2000)
pDH5-Del*sco*5113	pDH5 carrying the upstream sequence of *sco5113* (−1,480 to 147) and the downstream sequence of *sco5113* (1,663 to 2,929)	This study
pUC19	Cloning vector; Amp^r^	Promega
pUC19-*bldKB*-*wt* (AAG)	pUC19 carrying the complete *bldKB* (AAG) genes	This study
pUC19-*bldKB*-K425R (AGG)	pUC19 carrying the complete *bldKB* (AGG) genes	This study
pSET152	Integrating vectors; Apra^r^	Kieser et al. (2000)
pSET152-*bldKB*-*wt*	pSET152 carrying the complete *bldKB* (AAG) genes	This study
pSET152-*bldKB*-K425R	pSET152 carrying the complete *bldKB* (AGG) genes	This study
pET28a	*E. coli* overexpression vector; Kana^r^	Promega
pET28a-*bldKB*	N-terminal His_6_ fusion of *bldKB* cloned into pET28	This study
**Strains**		
*S. coelicolor*		
M145 (*SC*)	Prototroph	Kieser et al. (2000)
*SC*-∆*sco0988*	M145 (*SC*) with *sco0988* disrupted by pOJ260	This study
*SC*-∆*sco0988*/pWHM3-*ermE*	*SC*-∆*sco0988* carrying pWHM3-*ermE*	This study
*SC*-∆*sco0988*/pWHM3-*ermE*-*sco0988*	*SC*-∆*sco0988* carrying pWHM3-*ermE*-*sco0988*	This study
*SC-*Δ*bldKB*	M145 (*SC*) with *bldKB* excised by in-frame deletion	This study
*SC-ΔbldKB*/pSET152*-bldKB-wt*	*SC-*Δ*bldKB* carrying pSET152-*bldKB* (AAG)	This study
*SC-ΔbldKB*/pSET152*-bldKB-*K425R	*SC-*Δ*bldKB* carrying pSET152-*bldKB* (AGG)	This study
*S. lividans*		
TK24 (*SL*)	Prototroph	Kieser et al. (2000)
*SL*/pWHM3-*ermE*	TK24 carrying pWHM3-*ermE*	This study
*SL*/pWHM3-*ermE*-*sco0988*	TK24 carrying pWHM3-*ermE*-*sco0988*	This study
*E. coli*		
DH5α	F *rec*A *lac*ZM15	Promega
C43	F *omp*T *hsd*SB *gal dcm*	Miroux and Walker (1996)
C43/pET28a-*bldKB*	C43 carrying pET28a-*bldKB* for BldKB expression	This study
ET12567	*dam dcm hsd*S	Kieser et al. (2000)

### 2.4 Determination of growth and of RED and ACT production of native and genetically modified *SC* and *SL* strains

In order to quantify biomass yield as well as RED (undecylprodigiosin) and ACT (actinorhodin) production of native and genetically modified derivatives of *SC* and *SL* constructed in this study, 10^7^ spores of each strain were used to inoculate 200 mL of R2YE medium and grown for 72 h until stationary phase. Two hundred microliters of each culture was plated on cellophane discs deposited on the surface of 9 cm-diameter plates of R2YE solid medium in triplicates and the plates were incubated at 28°C. Half of the mycelium of each triplicate was collected every 12 h, from 24 h until 84 h of incubation, in order to establish a growth curve. Collected mycelial samples were dried at 55°C overnight and weighted. One quarter of the mycelium of each triplicate was used to assay intracellular RED and ACT concentrations and the agar medium below the last quarter was used to assay extracellular ACT concentrations as described previously but with slight modifications (Xu et al., 2010).

To assay intracellular RED, the mycelium was extracted by the addition of 1 mL of methanol then the mixture was acidified to pH 2–3 by addition of 1 mL HCl (1 M) whereas to assay intracellular ACT the mycelium was extracted by the addition of 1 mL of KOH (1 M). Samples were vortexed for 30 min at 4°C. To assay RED the optical density was measured at 530 nm against a blank constituted by methanol and HCl (1 M) (v/v:1/1) whereas to assay ACT the optical density was measured at 640 nm against a blank constituted by KOH (1 M).

To assay extracellular ACT, a quarter of each of the three R2YE agar plates was smashed and subjected to diffusion in 10 mL water at 4°C for at least 2 h. The mixture was then centrifuged and 10 mL of KOH (1N) was added to the collected supernatant. The solution was mixed by inversion, then 10 mL of HCL (3N) was added. The resulting mixture was incubated on ice for 10 min and the precipitated ACT was then collected by centrifugation. The supernatant was discarded and the ACT pellet was re-suspended in 1 mL KOH (1N). The optical density of the solution was measured at 640 nm against a blank constituted by KOH (1 M) in order to determine ACT concentration. In all cases a spectrophotometer (Molecular Devices, United States) was used to determine optical density.

### 2.5 Acetylome analysis

#### 2.5.1 Preparation of protein samples

In order to carry out comparative acetylome analysis of *SL*/pWHM3-*ermE* and *SL*/pWHM3-*ermE*-*sco0988*, 10^7^ spores of these strains were plated on cellophane discs laid on the surface of plates of R2YE solid medium. Approximately 400 mg of dry mycelium of each strain was collected from 10 plates after 24 h and 36 h of incubation at 28°C. At 24 h the strains did not produce any colored antibiotics whereas at 36 h, the strains just started to produce colored antibiotics. Cells were lysed by sonication and total proteins were quantified using the Bradford reagent (ABP Biosciences, United States). To maintain reducing state and stabilize proteins, DTT was added at a final concentration of 10 mM to each extracted protein samples (20 μg) that were incubated for 1.5 h at 37°C, and a sulfhydryl alkylating reagent iodoacetamide (IAA) was the added to prevent disulfide bond formation at a final concentration 50 mM and the mixture was incubated in the dark for 30 min at room temperature.

The proteins were digested with trypsin (trypsin: protein = 1:50, weight ratio) at 37°C overnight. The pH of the mixture was adjusted to pH ≤3 with trifluoroacetic acid (TFA) 10% (final concentration 0.1%). Each digested peptides sample was desalted on C18 Cartridges (Cat. No. 66872-U, Sigma) and lyophilized. The digested peptides present in 1.4 mL of precooled IAP buffer (50 mM MOPS/NaOH pH 7.2, 10 mM Na_2_HPO_4_, 50 mM NaCl) (PTMScan^®^, #9993) were enriched by immunoaffinity purification. To do so they were incubated for 1.5 h at 4°C, with a PTMScan^®^ Acetyl-Lysine Motif Antibody conjugated to protein A agarose beads (Cell Signaling Technology, United States). After centrifugation of the beads (2,000 × g, 30 s), the supernatant was discarded and the beads were washed thrice with 1 mL precooled IAP buffer then washed thrice again with precooled water. The washed beads were incubated with 40 μL TFA 10% (final concentration 0.15%) for 10 min at room temperature, then centrifuged (2,000 × g, 30 s). The supernatant containing the acetylated peptides was collected and this operation was repeated thrice. Desalted supernatants of the immunoprecipitated peptides utilizing C18 stage-tips (Cat. No. 22A00A001, Thermo) were prepared prior to LC-MS analysis (Nativio et al., 2020).

#### 2.5.2 LC-MS/MS analysis

A Q-Exactive HF/HFX mass spectrometer (Thermo Scientific^™^, United States) coupled with an Easy nLC (Proxeon Biosystems) (Thermo Scientific^™^, United States) was used for LC-MS/MS analysis (Chen et al., 2019). Using IntelliFlow technology, the peptides in formic acid (0.1%) buffer were loaded onto the reverse phase trap column (Acclaim PepMap100, 100 pm*2 cm, nanoViper C18; Thermo Scientific^™^, United States) on the C18-reversed phase analytical column (Easy Column, 10 cm long, 75 pm inner diameter, 3 pm resin; Thermo Scientific^™^, United States) and separated with a linear gradient of acetonitrile (84%) and formic acid (0.1%) at a flow rate of 300 nL/min (Cox et al., 2020). MS data were acquired in a positive ion mode by a data-dependent top10 method (Barkovits et al., 2020).

The MS raw data for each sample were combined and searched using the MaxQuant software for protein identification and quantitation analysis. The mass spectrometry proteomics data have been deposited to the ProteomeXchange Consortium^1^ via the iProX partner repository with the dataset identifier PXD041540.

#### 2.5.3 Bioinformatic analysis

The protein sequences of the selected differentially acetylated proteins were locally searched using NCBI BLAST+ client software (ncbi-blast-2.2.28+-win32.exe). Homologous sequences were found using InterProScan (Quevillon et al., 2005) and the functional ontological category of the proteins was established using Blast2Go.^2^ Enrichment analysis, based on the Fisher’s exact test (Shan and Gerstenberger, 2017), was carried out the considering the whole quantified proteins as background dataset. Benjamini–Hochberg correction (Benjamini and Hochberg, 1995) for multiple testing was used to adjust derived *p*-values. *p*-values inferior to 0.05 were considered as significant. Sequences including the six amino acids upstream and downstream of the modified site were used to predict motifs with the MEME software.^3^

### 2.6 Construction of a *Streptomyces coelicolor* M145 strain deleted for *bldKB/sco5113*

In order to delete *bldKB/sco5113* in *S. coelicolor* M145 (*SC*), two 1.5-kb DNA fragments flanking the *sco5113* coding region were amplified by PCR using primer pairs DelUp*sco5113*-*Hind*III-F/DelUp*sco5113*-*Xba*I-R and DelDown*sco5113*-*Xba*I-F/DelDown*sco5113*-*Kpn*I-R (Table 1) and *SC* genomic DNA as template. The resulting two PCR fragments were individually digested with the restriction enzymes present in the primers (Takara, Japan) and cloned into the pDH5 plasmid that carries thiostrepton resistance determinant (Kieser et al., 2000), using a triple ligation strategy. The resulting plasmid pDH5-Del*sco5113* suitable for *sco5113* deletion was transformed into *SC* protoplasts. The transformants were grown for three generations on plates of R2YE medium in absence of any selective pressure. Subsequently, spores resulting from the transformants were diluted and spread to yield isolated colonies. The thiostrepton resistance or sensitivity of the resulting sporulated colonies was determined by their replicate plating on R2YE and on R2YE with thiosptrepton at 50 μg/mL. The chromosomal structure of the transformants, that had lost the delivery plasmid and were only able to grown on R2YE, was assessed by PCR using the primer pair VerifDel*sco5113*-F and VerifDel*sco5113*-R (Table 1) and genomic DNA originating from these transformants. The mutants in which the successful in-frame deletion of *sco5113* was confirmed were called *SC-*Δ*bldKB*.

### 2.7 Complementation of *SC*-Δ*bldKB* by wild type and mutagenized versions of BldKB/SCO5113

In order to generate a mutagenized version of BldKB/SCO5113, a plasmid containing BldKB/SCO5113 ORF together with its native promoter was first constructed. To do so, a PCR fragment encompassing this region was amplified using the primer pair CP*sco5113*-*Eco*RI-F/CP*sco5113*-*Xba*I-R (Table 1) and *SC* chromosomal DNA as template. The resulting PCR product cut by *Eco*RI and *Xba*I was ligated into pUC19 to generate pUC19-*bldKB-wt* (AAG).

Subsequently, in order to generate mutagenized BldKB, the primer pairs K425R*sco5113*-F/K425R*sco5113*-R and K425Q*sco5113*-F/K425Q*sco5113*-R (Table 1), in which the 425th Lys codon (AAG) was replaced by an Arg codon (AGG) or by a Gln codon (CAG), were used to amplify the mutated fragment by PCR using pUC19-*bldKB-wt* as a template. The resulting PCR products were treated with *Dpn*I to digest the methylated parental DNA template, and subsequently transformed into *E. coli* DH5α competent cells. Plasmids isolated from various colonies were sequenced and the plasmids harboring the desired mutation (AAG/K to AGG/R) and (AAG/K to CAG/Q) were selected and named pUC19-*bldKB-*K425R and pUC19-*bldKB*-K425Q, respectively.

*bldKB-wt* (AAG), *bldKB-*K425R (AGG) and *bldKB-*K425Q (CAG) together with their native promoters were amplified by PCR using primer pair CP*sco5113*-*Eco*RI-F/CP*sco5113*-*Xba*I-R (Table 1) from pUC19-*bldKB-wt* (AAG), pUC19-*bldKB-*K425R (AGG) and pUC19-*bldKB*-K425Q (CAG), respectively. The resulting DNA fragments were ligated into the *Eco*RI and *Xba*I sites of pSET152 to generate pSET152-*bldKB*-*wt*, pSET152-*bldKB-*K425R and pSET152-bldKB-K425Q, respectively. These three plasmids were transformed into *SC*-Δ*bldKB* protoplasts to generate *SC*-Δ*bldKB*/pSET152-*bldKB*-*wt*, *SC*-Δ*bldKB*/pSET152-*bldKB-*K425R and *SC*-Δ*bldKB*/pSET152-*bldKB-*K425Q and the phenotype of the transformants was assessed for complementation.

### 2.8 *In vitro* acetylation of BldKB

In order to carry out *in vitro* acetylation of BldKB, recombinant His-tag fused BldKB was first expressed and purified in *E. coli* C to facilitate protein expression and purification (Miroux and Walker, 1996). To do so the whole *bldKB* coding region, except its termination codon, was amplified by PCR using primer pairs PE*sco5113-Eco*RI-F and PE*sco5113-Hind*III-R (Table 1). The resulting PCR fragments digested by *Eco*RI and *Hind*III (Takara, Japan) were cloned into the plasmid pET28a under the control of a LacI-like IPTG inducible promoter (Schifferli, 1995). In this plasmid, that carries a gene conferring resistance to kanamycin (Kan^r^), the cloned gene is fused to a DNA sequence translated as a 6-His-tag. The recombinant plasmid pET28a-*bldKB* was transformed into *E. coli* C43. *E. coli* C43/pET28a-*bldKB* was cultured in LB medium containing 25 μg/mL kanamycin at 37°C. When OD_600_ reached 0.4–0.5, IPTG was added at a final concentration of 0.5 mM and induction was allowed for 5–6 h. The cells were collected by centrifugation, washed in PBS (137 mM NaCl, 3 mM KCl, 10 mM Na_2_HPO_4_, 2 mM KH_2_PO_4_, pH = 7.4) and sonicated to obtain homogenous cell crude extracts. His-tag fused BldKB was purified to near homogeneity using Ni-NTA column according to the manufacturer’s instructions (Sangon Biotech, Shanghai Co., Ltd.).

However, since the acetyltransferase SCO0988 is a membrane protein, that could not be purified in *E. coli*, crude extracts of *SL*/pWHM3-*ermE* and *SL*/pWHM3-*ermE*-*sco0988* had to be prepared as described in You et al. (2019) in order to carry out the acetylation assays. To do so, these two strains were cultivated in R2YE liquid medium until stationary phase and their mycelium was collected by centrifugation, washed in PBS, re-suspended in sonication buffer (50 mM Tris-HCl pH 8.0, 10% glycerol, 0.1 mM EDTA, 1 mM dithiothreitol) containing a deacetylase inhibitor cocktail (Beyotime, Shanghai) and sonicated in order to obtain a homogeneous solution that was centrifuged to remove cell debris. The protein concentration of each supernatant was determined using the Bradford reagent (Catalogue No. 500-0006; Bio-Rad) and adjusted to 500 μg/mL for further uses. In the acetylation assay, 5 μg of purified six-histidine-tagged BldKB (His_6_-BldKB) and 1 μg of the crude extract of either *SL*/pWHM3-*ermE* or *SL*/pWHM3-*ermE*-*sco0988* were mixed together with 20 μM of acetyl-CoA in HAT buffer (50 mM Tris-HCl pH 8.0, 10% glycerol, 0.1 mM EDTA, 1 mM dithiothreitol) and incubated at 30°C for 3 h. Control was carried out in parallel with the reaction mix containing BldKB alone in presence of AcetylCoA.

At the end of the incubation period, His_6_-BldKB present in the reaction mixture was purified using Ni-NTA column following the manufacturers’ instructions. The collected fractions were separated on 12% SDS-PAGE and transferred on a polyvinylidene fluoride (PVDF) membrane that was incubated with antibodies against acetylated lysine (Cat. No. ICP0380, Runzekang, Beijing). Visualization of the acetylated protein was achieved by the addition of secondary antibody (HRP Goat Anti-Rabbit IgG) and a solution containing 3,3′-Diaminobenzidine (DAB) (Cat. No. PK10005, Proteintech) the substrate of HRP as described in Kim et al. (2013).

## 3 Results

### 3.1 SCO0988 positively regulates specialized metabolism and morphological differentiation in *Streptomyces coelicolor* and *Streptomyces lividans*

SCO0988 belongs to the Gcn5-related N-acetyltransferase (GNAT) (Pfam00583) superfamily whose carboxyl terminal end catalyzes the transfer of the acetyl moiety of acetyl-CoA to the ε-amino group of a lysine residue (Vetting et al., 2005). The Kyte and Doolite hydropathic profile (Kyte and Doolittle, 1982) of this protein suggested that it is a membrane protein with multiple trans membrane segments (Supplementary Figure S1). The level of expression of *sco0988* was shown to increase with time indicating that it might regulate late developmental processes such as morphological differentiation and secondary/specialized metabolism (Supplementary Figure S2) (Zhang et al., 2020).

**Figure 1:**
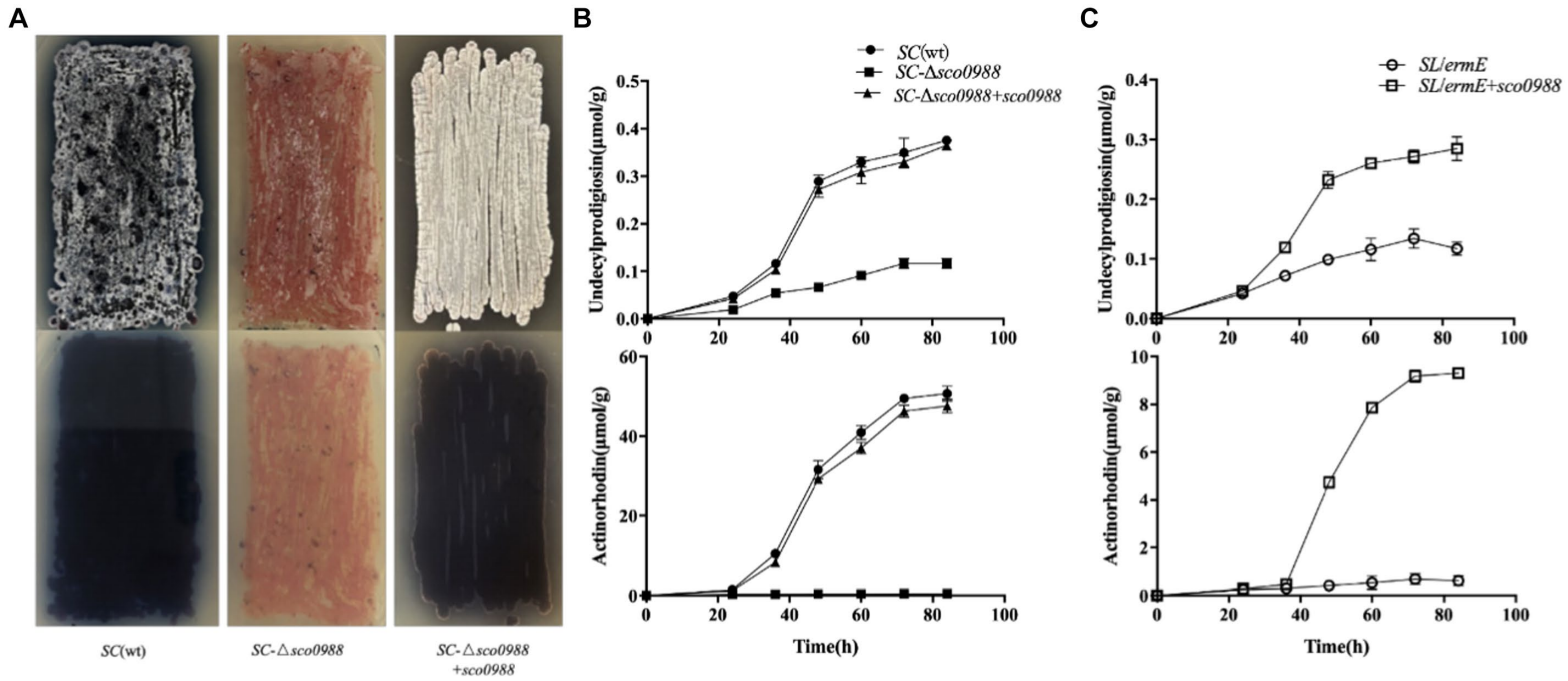
Phenotype resulting from the deletion of *sco0988* in *S. coelicolor* M145 and from the over-expression of *sco0988* in *S. lividans*. **(A)** Front/back pictures of lawns of the original strain *S. coelicolor* M145, of the mutant deleted for *sco0988* and of this mutant complemented by *sco0988* carried the plasmid pWHM3-*erm*E-*sco0988*. The strains were grown on solid R2YE medium for 48 h. **(B)** Quantitative analysis of RED and ACT production of the three *S. coelicolor* strains mentioned above. **(C)** Quantitative analysis of RED and ACT production of *S. lividans* TK24 and of this strain transformed by the plasmid pWHM3-*erm*E-*sco0988*. For **(B,C)** the strains were grown in liquid R2YE medium for 72 h at 28°C under constant shaking of 220 rpm.

In order to determine whether this acetyltransferase has an impact on these processes in the strong antibiotic producer, *SC*, a *sco0988* disruptive mutant of *SC*, *SC*-Δ*sco0988* was constructed. The phenotypes of the mutant, and of the *wt* strain are shown in Figure 1. The *sco0988* deletion mutant had a slightly slower growth rate than the original strain (Supplementary Figure S3) and was characterized by a strong inhibition of both antibiotic production and sporulation (Figure 1A). Quantitative analysis demonstrated a 2/3 reduction of RED production and a complete abolition of ACT biosynthesis in *SC*-Δ*sco0988* compared to the original strain (*p* < 0.05) (Figure 1B). The complementation of the *SC*-Δ*sco0988* by *sco0998* carried by the high copy number plasmid pWHM3 (Bibb et al., 1985) restored growth (Supplementary Figure S3) as well as RED and ACT production to the level of the original strain whereas sporulation was enhanced compared to that of the *wt* strain (Figures 1A,B).

In order to determine whether this acetyltransferase has an impact on these processes in the weak antibiotic producer, *SL*, *sco0988* carried by pWHM3 was over-expressed in this strain. This over-expression led to a two-fold increase of RED production and the originally undetectable ACT production became now detectable (Figure 1C). The over-expression of *sco0988* had no impact on the growth rate of the strain that was similar to that of the strain containing the empty plasmid (data not shown). These results clearly demonstrated that SCO0988 has a positive impact on both antibiotic production and morphological differentiation in these two model *Streptomyces* species.

### 3.2 Global analysis of acetylome of *Streptomyces lividans* TK24 reveals the acetylation of numerous proteins involved in diverse cellular processes

**Figure 2:**
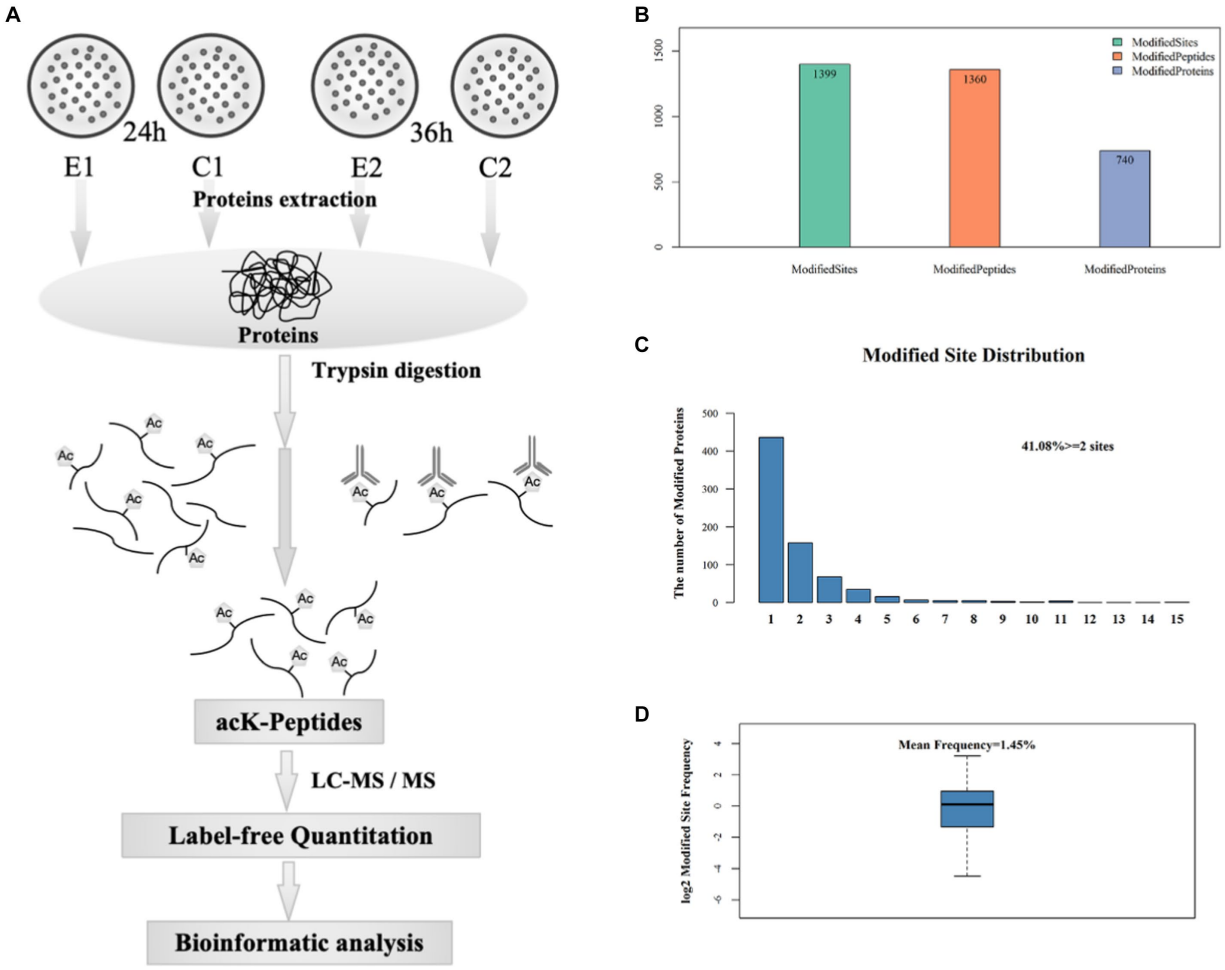
Preparation and analysis of acetylated peptides. **(A)** Workflow used to purify acetylated peptides. **(B)** Acetylome analysis: number of sites, peptides and proteins with acetyl modifications. **(C)** Distribution of the number of acetylated proteins bearing 1 to 15 acetylated sites. **(D)** Occurrence frequency of lysine acetylated sites.

In order to gain an insight into the biological processes affected by the over-expression of *sco0988* in *S. lividans* TK24, a comparative analysis of the acetylome of the control strain (*SL*/pWHM3-*ermE*) and of the strain overexpressing *sco0988* (*SL*/pWHM3-*ermE*-*sco0988*) was carried out. Acetylated lysine (acK) peptides were purified by immunoaffinity-based acetyl-lysine peptide enrichment, identified and quantified in both strains by high resolution mass spectrometry. An overview of the experimental procedures used is provided in Figure 2A.

A total of 1,399 acetylated lysine sites were discovered in 740 proteins (Figure 2B) that represented approximately 10% of *S. lividans* TK24 proteome (Rückert et al., 2015). 59% of these proteins, contained a single acetylated site, 21% had two acetylated sites and 9% contained three acetylated sites (Figure 2C). The average frequency of lysine acetylation was of 1.45 acetylated residue in 100 amino acids (Figure 2D). Detailed information concerning all acetylated peptides and the corresponding proteins is provided in Supplementary Table S1. A total of 643 statistically differentially acetylated peptides (*p* < 0.05) at 24 h and 36 h is shown in Supplementary Table S2. Considering that 54 of them were present at the two time points, the amount of the acetylated peptides is thus 589 (643-54). Among these 589 acetylated peptides, 177 (30.05%) were more acetylated while 358 (60.78%) were less acetylated in the strain overexpressing *sco0988* than in the control strain. These observations suggested that in *SL* the pool of acetylCoA is limited and that the over-expression of *sco0988* consumes acetylCoA and thus limits the acetylation of the specific targets of other acetyltransferases. Interestingly, 92 acetylated peptides were found exclusively present in the strain over-expressing *sco0988* whereas 159 acetylated peptides were only present in the control strain (Supplementary Tables S1, S2). The proteins over-acetylated or exclusively acetylated in *SL*/pWHM3-*ermE*-*sco0988* can thus be considered as potential specific SCO0988 targets.

**Figure 3:**
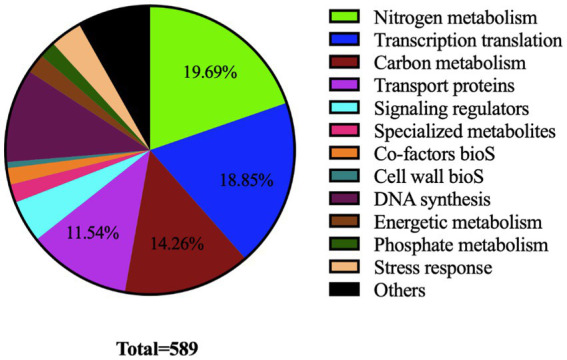
Pie chart showing ontological classification of proteins acetylated by the acetyltransferase SCO0988 in *S. lividans*.

Gene Ontology (GO) terms and KEGG pathway enrichment analysis of differentially acetylated proteins (DAPs) was performed with Applied Protein Technology Co., Ltd (Shanghai, China). The ontological classifications, determined by Blast2Go (see text footnote 2) and Interpro scan,^4^ are shown in Figure 3. A large proportion of DAPs (>60%) were classified as involved in nitrogen (19.6%) and carbon (14.93%) metabolisms, transcriptional and translational processes (18.97%) as well as transport (12.44%).

### 3.3 Motif analysis of lysine-acetylated sites

**Figure 4:**
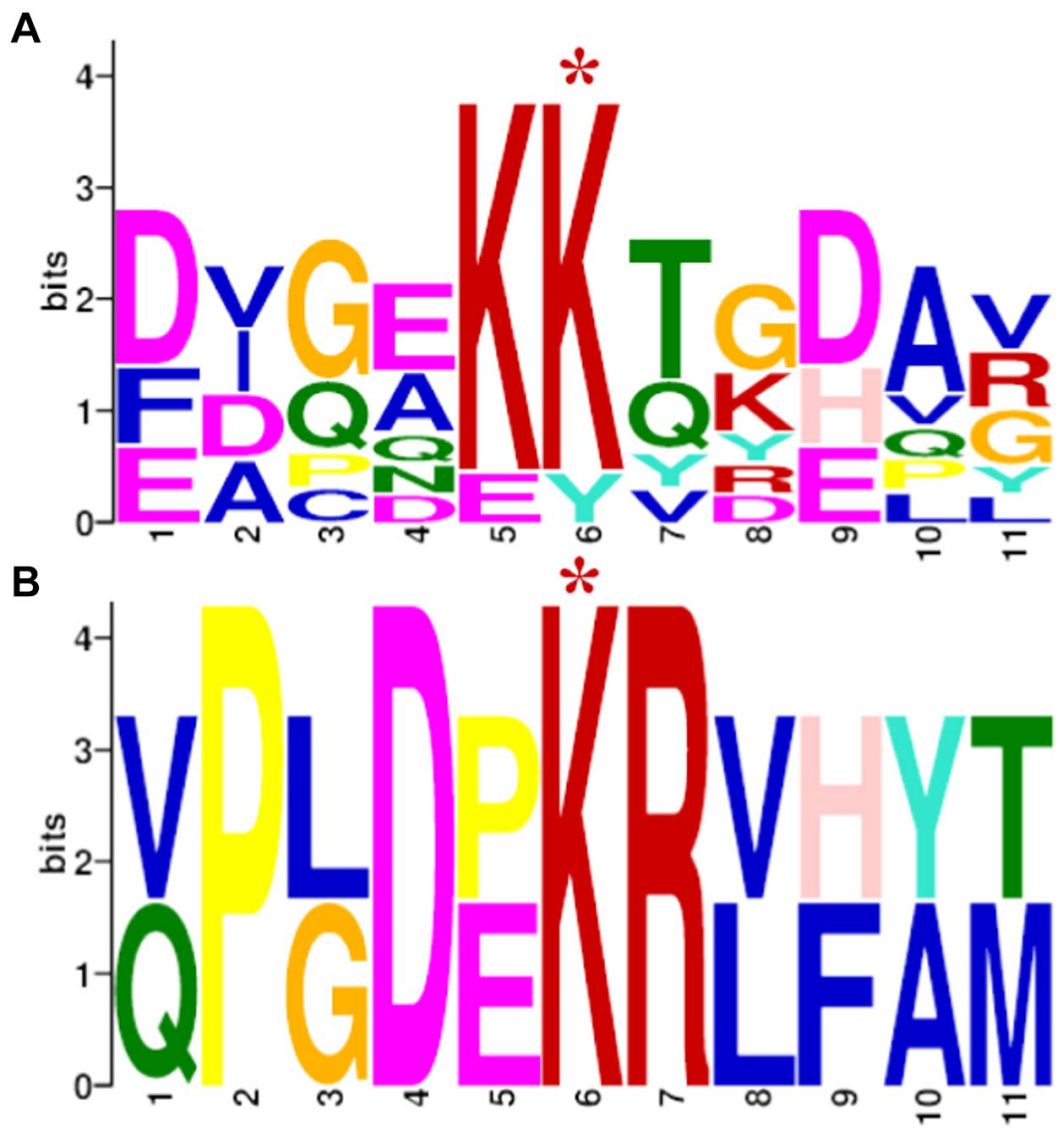
Probable sequence motifs of acetylation sites identified using the MEME software. These sites consist of 5 residues present on each side of the modified lysine residue signaled by a red asterisk. **(A)** Sequence motifs of the 159 acetylated lysine sites identified exclusively in the control strain. **(B)** Sequence motifs of the 92 acetylated lysine sites identified exclusively in the *S. lividans* strain over-expressing SCO0988.

In order to identify a possible consensus acetylation motif of SCO0988, the frequency of amino acids present from position −5 to +5 around the 92 lysines exclusively acetylated in the strain over-expressing SCO0988 were determined using the MEME program, and similarly, the frequency of amino acids presents from position −5 to +5 around 159 lysines exclusively acetylated in the control strain were determined using the MEME program. The results shown in Figure 4 demonstrated that, as expected, the consensus of the sites acetylated by SCO0988 was different and also far less diverse than the consensus of the other acetylated sites that are likely to be acetylated by multiple acetyltransferases other than SCO0988. In the latter the central pair KK is highly conserved but is replaced by KR in the SCO0988 consensus. K and R are both basic amino acids. In the SCO0988 consensus the positions 2, 3, 5 and 6 are highly conserved and are P, D, K and R, respectively and in the other positions only 2 alternative amino acids are present.

### 3.4 The extracellular solute-binding protein BldKB shows an increase in its acetylation level upon *sco0988* overexpression

BldKB/SCO5113 is the extracellular solute-binding protein of the ABC transporter BldKABCDE (SCO5112-SCO5116) also constituted of BldKA and BldKC (SCO5112 and SCO5114) two integral membrane proteins, BldKD/SCO5115/intracellular ATPase subunit and BldKE/SCO5116/ATP-binding protein. This ABC transporter is involved in the up-take of the BLD261 oligopeptide that is a signaling molecule of the quorum sensing pathway (Willey et al., 1993). The up-take of this signaling molecule by the BldK transporter triggers the expression of genes of the “bald” signaling cascade that controls positively antibiotic production as well as aerial mycelium formation and sporulation in *SC* (Nodwell et al., 1999). Since, 4 of the 7 acetylated lysine (acK) peptides detected in BldKB/SCO5113 showed a great increase in their acetylation level upon *sco0988* overexpression, with the Lys425 showing the largest increase (Supplementary Table S2), BldKB was selected for further analysis.

### 3.5 SCO0988 acetylates BldKB *in vitro*

**Figure 5:**
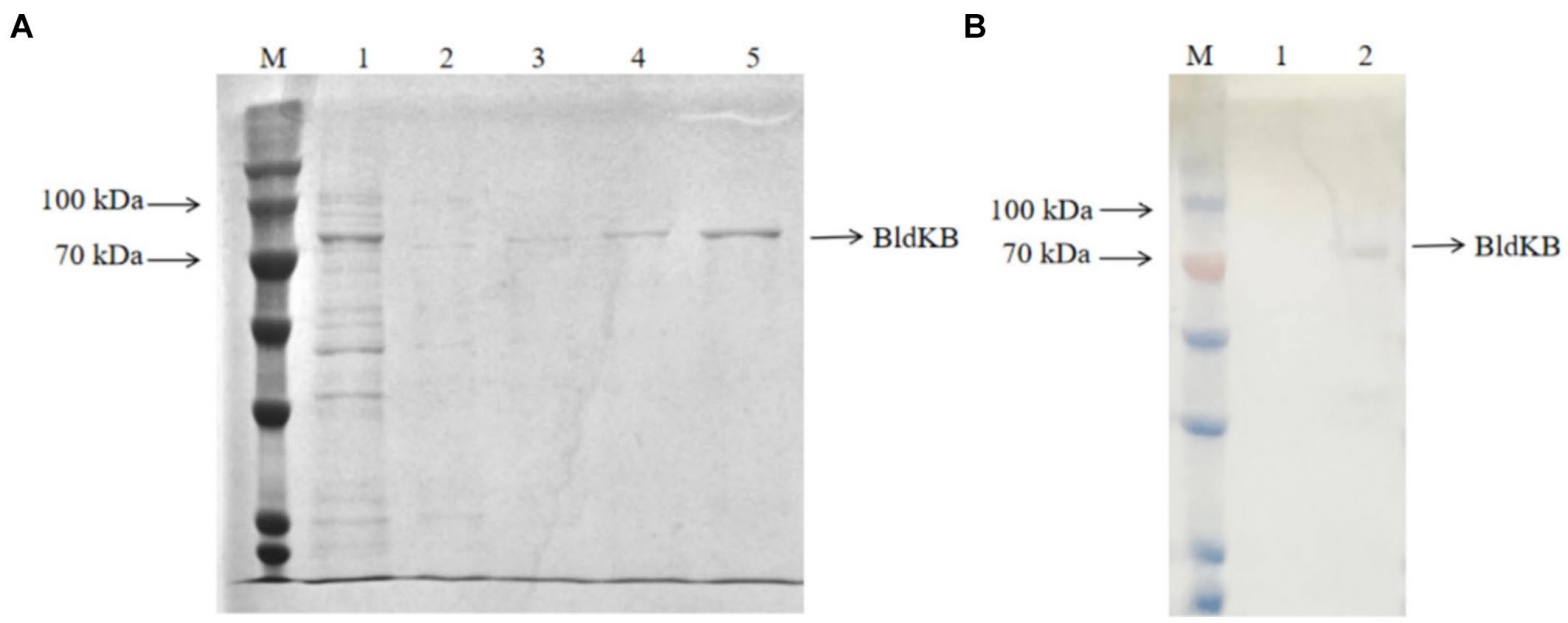
*In vitro* acetylation assay of BldKB. **(A)** SDS-PAGE analysis of His_6_-BldKB purified using *E. coli*/pET expression system. Lane M: marker (Cat. No. PMK06-01, Biocomma); lane 1: flow through; lanes 2–3: elution with 100 mM imidazone; lanes 4–5: elution with 250 mM imidazone. **(B)** Western blot analysis of BldKB acetylation in presence of cell crude extracts of *S. lividans*/pWHM3 (lane 1) and *S. lividans*/pWHM3-*ermE*-*SCO0988* (lane 2) using antibodies against acetylated lysine.

In order to determine whether SCO0988 was able to acetylate BldKB *in vitro*, recombinant His_6_-BldKB was expressed in *E. coli* and purified (Figure 5A). Since the membrane protein SCO0988 formed inclusion bodies when *E. coli*/pET expression system was used (data not shown), cell crude extracts from *S. lividans* over-expressing *sco0988* were prepared for the acetylation assay. Western blot probed with an antibody against acetylated lysine clearly demonstrated an intensified band corresponding to acetylated BldKB in the reaction carried out in the presence of crude extract of *SL*/pWHM3-*ermE*-*sco0988* (Figure 5B) but not in that carried out in the presence of crude extract of *SL*/pHWM3-*ermE* (Figure 5B). No acetylation was observed in the control assays with BldKB alone in presence of AcetylCoA (data not shown). These data thus demonstrate the acetylation of BldKB by SCO0988.

### 3.6 Is the acetylation of the lysine 425 of BldKB necessary for its function?

**Figure 6:**
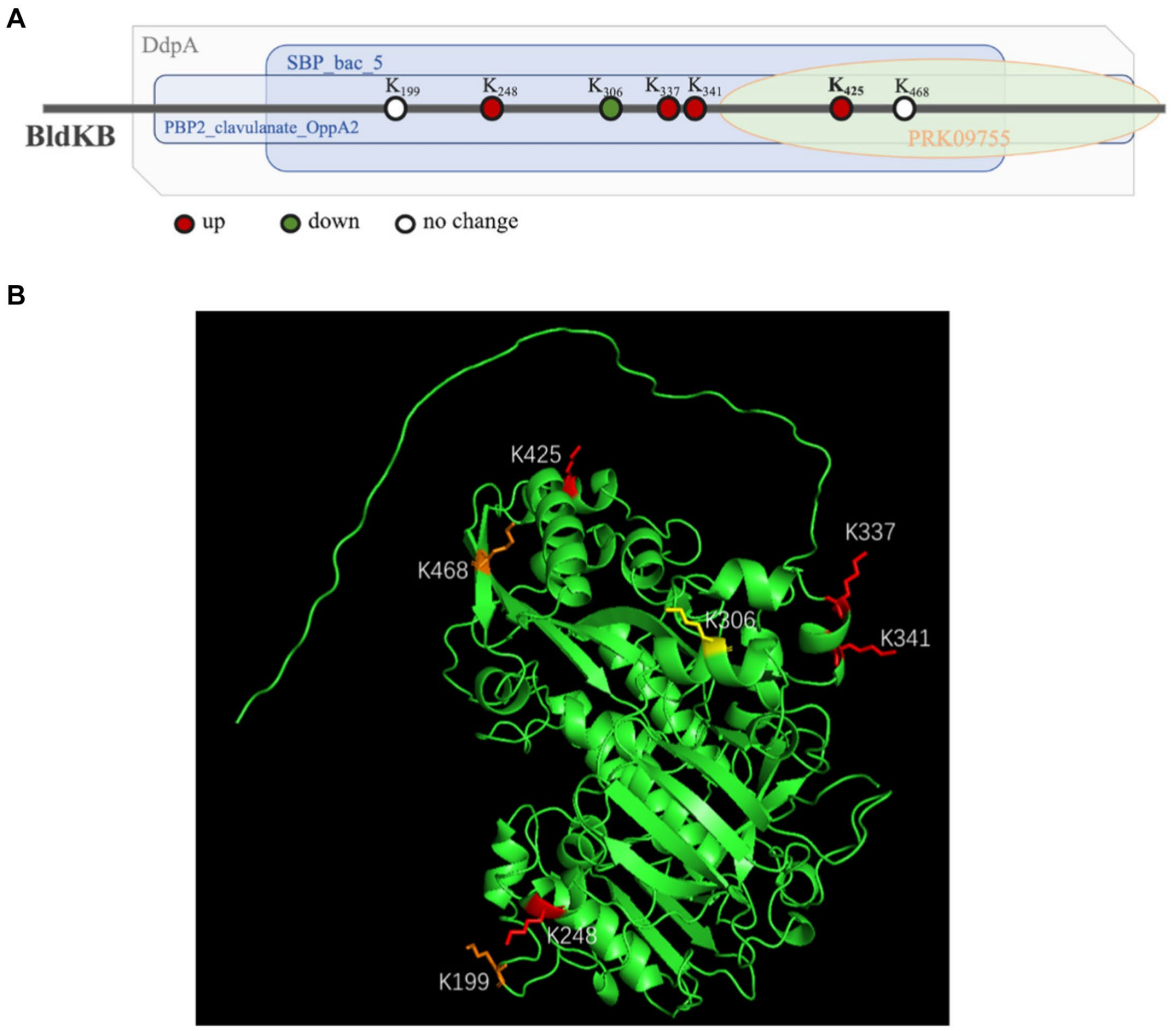
Structure of the BldKB protein. **(A)** Mapping of the four domains identified by InterProScan in the BldkB protein and positioning of the acetylated lysines (K). **(B)** Positions of the seven acetylated lysines of BldKB (K199, K248, K337, K341, K425, K468 and K306) on its three-dimensional structure predicted by AlphaFold and PyMol. The four Lys over-acetylated (K248, K337, K341 and K425), the two Lys with un-changed level of acetylation (K199 and K468) and the Lys under-acetylated (K306), in the strain SL/pWHM3-*ermE*-sco0988, are represented in red, orange and yellow, respectively.

**Figure 7:**
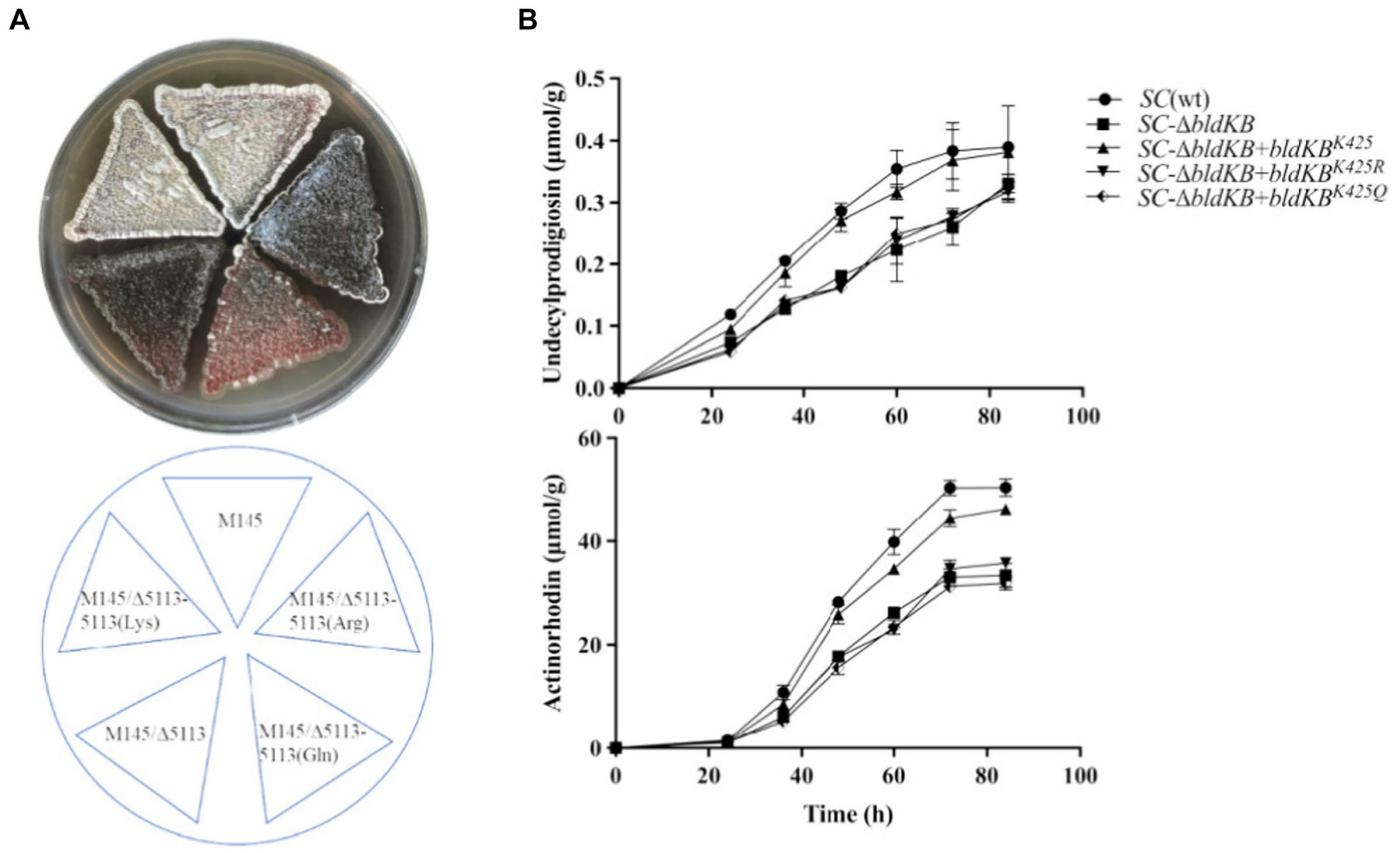
Phenotypes of the genetically modified strains under study. **(A)** Front and back pictures of lawns of *S. coelicolor* M145, of the *bldKB* deleted strain (*SC*-Δ*bldKB*) and of this mutant strain complemented by *bldKB^K425^* (AAG/Lys), *bldKB^K425R^* (AGG/Arg) or *bldKB^K425Q^* (CAG/Gln). The strains were grown on solid R2YE medium for 72 h. **(B)** Quantitative analysis of RED and ACT productions by the various strains grown in liquid R2YE medium.

According to NCBI’s Conserved Domain Database (CDD), four structural domains could be identified in BldKB: a periplasmic component DdpA-like (57–592), an oligopeptide binding domain OppA2-like (75–582), an extracellular solute-binding domain SBP_bac_5-like (120–516) and a substrate-binding domain PRK09755 (356–599) (Figure 6A).^5^ All four domains are critical for the BldKB function. Seven acetylated lysine sites were detected in BldKB and among them four (K/248, K/337, K/341 and K/425) were over-acetylated upon *sco0988* over-expression (Supplementary Tables S1, S2 and Figure 6). Interestingly K_425_, the most sensitively acetylated Lys upon SCO0988 over-expression, is present in the four domains detected in BldKB (Figure 6). In order to determine whether K_425_ was important the function and/or the acetylation of BldKB, a *SC* strain disrupted for *bldKB*, *SC-*Δ*bldKB*, was first constructed. *SC-*Δ*bldKB* had a similar growth pattern as the original strain (Supplementary Figure S4) but its RED and ACT production levels were reduced, being 32.03 and 34.53% lower, respectively, than that of the original strain, at 72 h (Figures 7A,B). In contrast, morphological differentiation and sporulation seemed totally abolished in *SC*-Δ*bldKB*. These observations are consistent with those reported in previous studies (Nodwell et al., 1996; Nodwell and Losick, 1998; Park et al., 2005). Subsequently, variants of BldKB in which Lys^425^ was substituted by Arg/R or Gln/Q were constructed. Arg/R was chosen since it cannot be acetylated and Gln/Q was chosen since many reports in the literature mentioned that it mimics acetylated Lys (Kamieniarz and Schneider, 2009; Okada et al., 2021; Morse et al., 2023). When *bldKB* (K425R) and *bldKB* (K425Q) were introduced into *SC*-Δ*bldKB*, complementation did not occur since ACT and RED production as well as sporulation were not restored to wild type level (Figures 7A,B). In contrast, these features were restored to wild type level when the native *bldKB* gene was introduced into *SC*-Δ*bldKB*. These results indicated that Lys^425^ is crucial for BldKB function and that the replacement of Lys^425^ by Arg or Gln greatly alters BldKB function. Unfortunately, these results were inconclusive concerning the impact of acetylation on BldKB function (see discussion).

## 4 Discussion

In bacteria Lys acetylation in proteins can result from two different processes: a chemical process using the high energy metabolites acetyl phosphate and acetylCoA as acetyl donor and an enzymatic process resulting from a catalytic reaction between an acetyltransferase, an acetyl donor (usually acetylCoA), and a lysine amino acid acceptor present in a protein substrate. Interestingly, Interestingly, non-enzymatic and acetyltransferase-dependent acetylation sites are usually different, suggesting that these two acetylation mechanisms play distinct roles in the post-translational modifications of bacterial proteins (Christensen et al., 2018, 2019). The “regulatory importance” of enzymatic protein acetylation has been demonstrated in many microbial processes including primary and specialized metabolisms, DNA replication, chemotaxis, virulence etc.… (Galdieri et al., 2014; Menzies et al., 2016) whereas a report suggested that AcP-driven acetylation have little functional consequences at least in *E. coli* (Schastnaya et al., 2023).

It is noteworthy that the impact of protein acetylation on the activity of target proteins remains largely unknown in Actinobacteria of the *Streptomyces* genus that are well known producers of bio-active specialized metabolites of great economic importance. Still, some proteins directly involved in the biosynthesis of bio-active specialized metabolites have been shown to be acetylated in *Streptomyces roseosporus* (Liao et al., 2014) and *Streptomyces griseus* (Ishigaki et al., 2017). The acetylation, on Lys70, of the deoxysugar epimerase StrM of the streptomycin biosynthetic pathway of *S. griseus*, lowers its activity and this results into a reduced streptomycin production (Ishigaki et al., 2017). Furthermore, GlnR, the central response regulator of nitrogen assimilation that governs the expression of numerous genes involved into N-assimilation in most Actinomycetes species (Amin et al., 2016), activates the expression of the Gcn5-type lysine acetyltransferase AcuA and of the NAD^+^ dependent deacetylase SrtN in *Saccharopolyspora erythraea* (You et al., 2017). These enzymes are involved in the acetylation of 3 AMP-forming acetyl-CoA synthetases and has a negative impact on their activity (You et al., 2016). The activity of an acetoacetyl-CoA synthetase (Martín et al., 2021) was also shown to be controlled negatively by O-serine and Nε-lysine acetylation in *SL* (VanDrisse and Escalante-Semerena, 2018). In *SC*, the function of the regulator GlnR/SCO4159, is modulated by post-translational modifications including phosphorylation of Ser/Thr residues and acetylation of Lys residues. GlnR was shown to be acetylated on 4 Lys residues when *SC* is grown in defined a media and this acetylation was independent of N-concentration. In contrast, GlnR was acetylated on a single Lys residue upon growth in a complex N-rich medium. However acetylation seems to have little influence on the formation of GlnR-DNA complex whereas phosphorylation inhibits the binding of GlnR to its targets genes (Sun et al., 2020a).

In our study, the comparative analysis of the acetylomes of the native strain of *S. lividans* and of the *S. lividans* strain overexpressing the acetyltransferase SCO0988 revealed a total of 1,399 acetylation sites in 740 proteins. Interestingly, among these 1,399 acetylated peptides detected, 589 (42.10%) are showing significant differences in their acetylation level between the two strains (ratio +/− 2 with *p* < 0.05) (Supplementary Table S2). Our study revealed that 92 proteins were exclusively acetylated and 118 were over-acetylated in the strain over-expressing SCO0988. These proteins can thus be considered as specific SCO0988 targets. We focused our study on one of the proteins extensively over-acetylated in the strain over-expressing SCO0988. This protein is the extracellular solute-binding protein BldKB of the oligopeptide transporter (BldKABCDE/SCO5112-SCO5116). This protein was chosen since it was shown to be acetylated on 7 different Lys residues. Four of these Lys (K_248_, K_337_, K_341_ and K_425_) were over-acetylated, in the strain over-expressing *sco0988* whereas the acetylation level of K_199_ and K_468_ was unchanged and that of K_306_ was reduced (Figure 6). Lys are positively charged residues and their acetylation that neutralize these positive charges might be important for the BldKB function. Indeed, the BLD261 oligopeptide transported by the BldK ABC transporter is likely to be positively charged as its NH^2+^ groups could be ionized into NH^3+^. Since charges of the same sign repel each other, the similar electrostatic charges of these two “partners” might prevent their interaction whereas the neutralization of the positive charge of Lys by acetylation would allow this interaction. Considering that it was the acetylation level of K_425_ that showed the greatest increase in the SCO0988 over-expressing strain, we decided to replace K_425_ by an Arg/R or a Gln/Q residue, in order to determine whether these substitutions had an impact on the function and/or on the acetylation of BldKB. These residues were chosen since R cannot be acetylated whereas Q is expected to mimic acetylated K (Kamieniarz and Schneider, 2009; Okada et al., 2021; Morse et al., 2023). The native BldKB as well as the mutated versions of BldKB, BldKB^K425R^ and BldKB^K425Q^, were used to complement the *bldkB* deletion mutant of *SC*. Our results revealed that only the native gene was able to restore the phenotype of the original strain indicating that Lys^425^ was critical for BldKB function and that its replacement by Arg or Gln deeply alters BldKB function. These replacements might alter the functionality, the stability or the proper folding of the protein. Alternatively, one cannot exclude that the replacement of K_425_ by Q does not effectively mimic acetylated K_425,_ in our specific context.

In conclusion, we wish to stress that our study demonstrated that acetylation could constitute a valuable tool to enhance the expression of already known or of cryptic biosynthetic pathways of specialized metabolites present in the *Streptomyces* genomes in order to discover most needed novel antibiotics to face the worrying emergence and rapid spreading of antibiotic-resistant pathogens. However, considering the multiplicity of SCO0988 targets, including 10 proteins annotated as being involved in signalization and regulation, that are exclusively acetylated in the strain over-expressing SCO0988 (Supplementary Table S2), the lack of acetylation of these regulatory proteins and possibly that of other proteins, besides BldKB, might contribute to the reduction of antibiotic production and to the inhibition of morphological differentiation and sporulation of the *sco0988* deletion mutant of *SC*.

## Data availability statement

The original contributions presented in the study are included in the article/Supplementary material, further inquiries can be directed to the corresponding authors.

## Author contributions

YB: Data curation, Formal analysis, Investigation, Methodology, Software, Writing – original draft. HA: Data curation, Formal analysis, Investigation, Methodology, Writing – original draft. ZC: Conceptualization, Formal analysis, Investigation, Methodology, Writing – original draft. ZX: Data curation, Formal analysis, Investigation, Software, Writing – original draft. YD: Data curation, Investigation, Methodology, Writing – original draft. YR: Formal analysis, Investigation, Methodology, Writing – original draft. RW: Formal analysis, Investigation, Methodology, Writing – original draft. XL: Data curation, Investigation, Methodology, Writing – original draft. JG: Conceptualization, Data curation, Formal analysis, Methodology, Resources, Software, Validation, Writing – review & editing. RH: Conceptualization, Data curation, Formal analysis, Methodology, Software, Supervision, Validation, Writing – review & editing. M-JV: Conceptualization, Data curation, Formal analysis, Supervision, Validation, Visualization, Writing – original draft, Writing – review & editing. DX: Conceptualization, Funding acquisition, Investigation, Project administration, Supervision, Validation, Visualization, Writing – original draft, Writing – review & editing.

## Footnotes

^1^https://proteomecentral.proteomexchange.org/cgi/GetDataset?ID=PXD041540

^2^https://www.blast2go.com/

^3^http://meme-suite.org/index.htm

^4^http://www.ebi.ac.uk/InterProScan/

^5^https://www.ncbi.nlm.nih.gov/Structure/cdd/wrpsb.cgi?INPUT_TYPE=precalc&SEQUENCE=1532202
