# Supplementary material for: The acetyltransferase SCO0988 controls positively specialized metabolism and morphological differentiation in the model strains Streptomyces coelicolor and Streptomyces lividans
Source: Front Microbiol. 2024 Jul 24;15:1366336. doi: 10.3389/fmicb.2024.1366336 (PMC11303876; doi:10.3389/fmicb.2024.1366336)
Supplement: Supplementary file 1 [file Table_1.DOCX]

Supplementary Material

# Supplementary Data

Article title: The acetyltransferase SCO0988 controls positively specialized metabolism and morphological differentiation in *Streptomyces coelicolor* and *Streptomyces lividans*

# Supplementary Figures and Tables


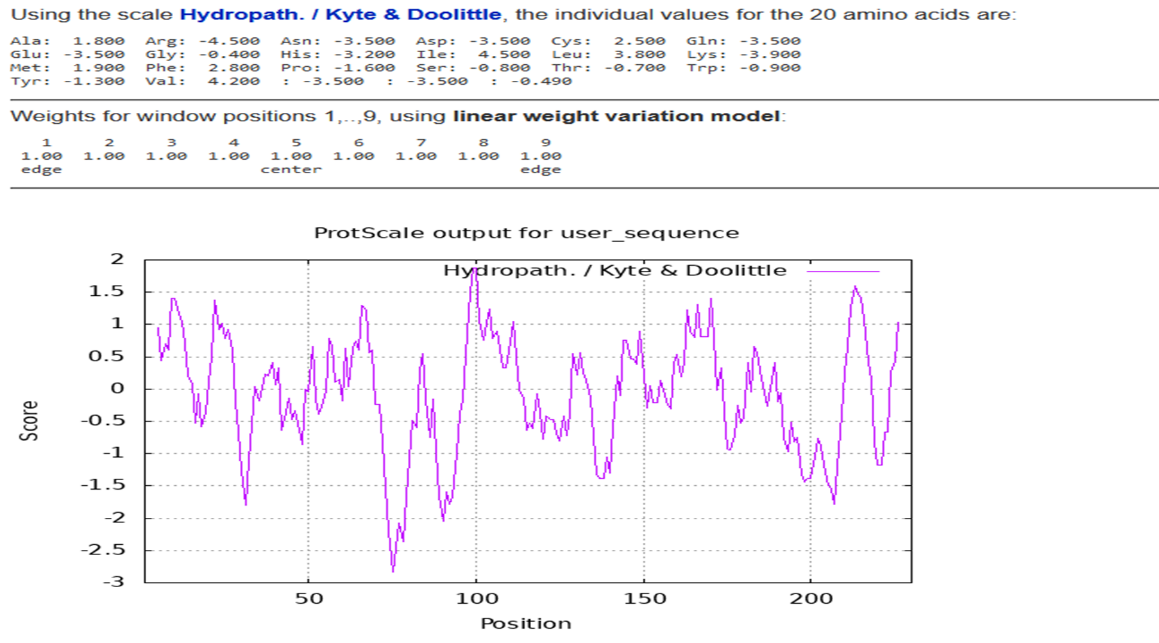


**Fig. S1.** Kyte and Doolite hydropathic profile of SCO0988 showing numerous hydrophobic trans-membrane segments.


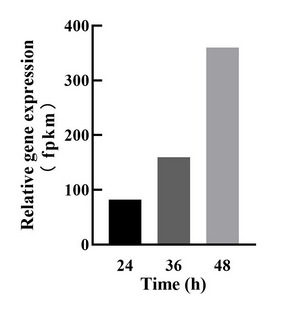


**Fig. S2.** Relative level of *sco0988* mRNA expression in *S. coelicolor.* These data were extracted from Frontiers in Microbiology (2020), 11: 1399, doi: 10.3389/fmicb.2020.01399.


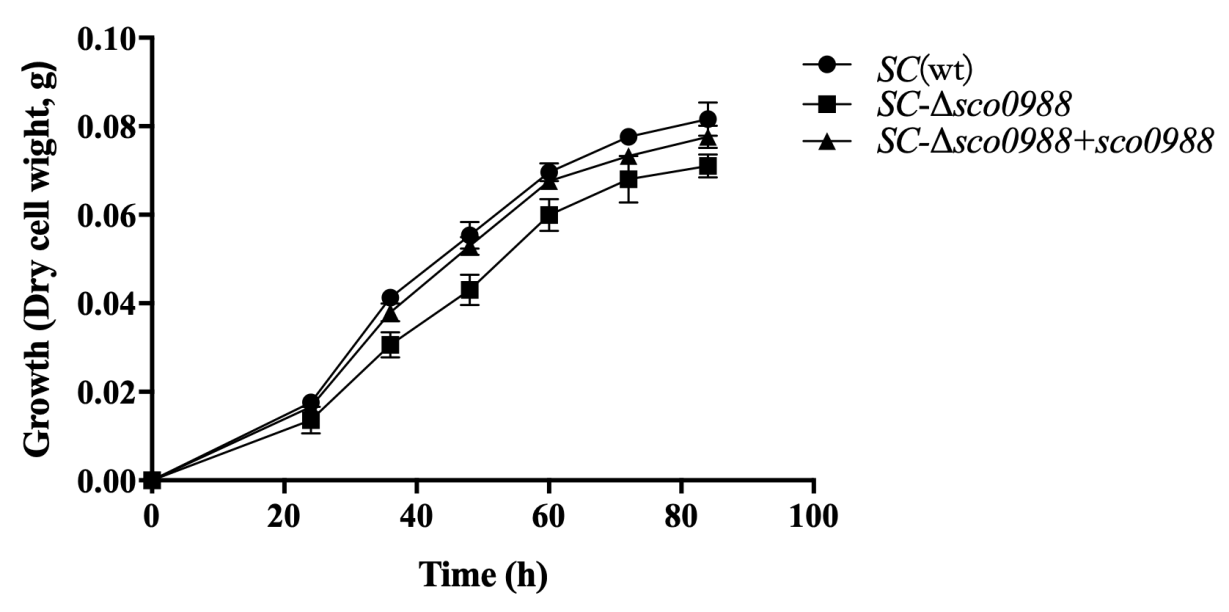


**Fig. S3.** Growth curves of the wild-type strain of *S. coelicolor* (*SC*), of the *sco0988* inactivated strain (*SC*-Δ*sco0988*) and of *SC*-Δ*sco0988* complemented with pWHM3-*ermE*-*sco0988* (*SC*-Δ*sco0988*+*sco0988*)


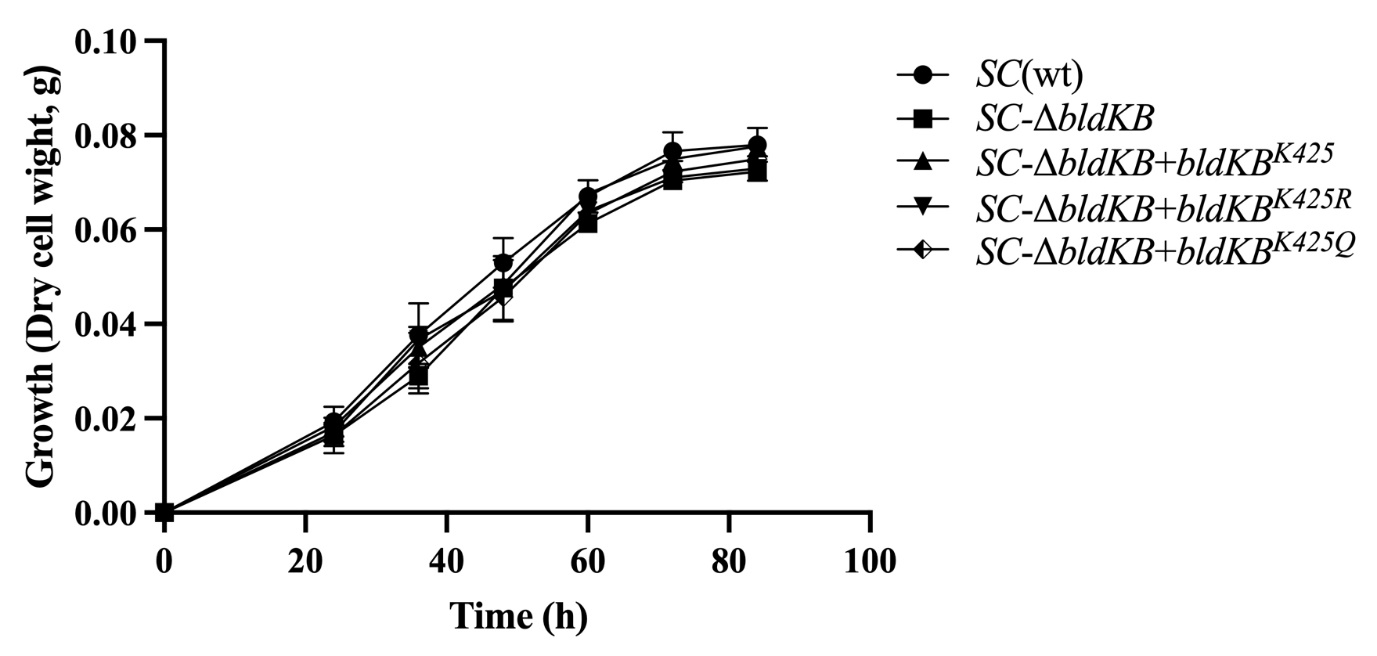


**Fig. S4.** Growth curves of the wild-type strain of *S. coelicolor* (*SC*), of the *bldKB* inactivated strain (S*C*-Δ*bldKB*) and of *SC*-Δ*bldKB* complemented with pSET152-*bldKB^K425^* yielding SC-Δ*bldKB*+*bldKB^K425^*, with pSET152-*bldKB^K425R^* yielding SC-Δ*bldKB*+*bldKB^K425R^* or with pSET152-*bldKB^K425Q^* yielding SC-Δ*bldKB*+*bldKB^K425Q^*.

**Table S1** **List of detected acetylated peptides and corresponding proteins.**

Please find the attached excel document for details.

**Table S2 589 differentially acetylated peptides.**

Please find the attached excel document for details.

Bright orange: Peptides identified exclusively in the strain over-expressing SCO0988.

Light orange: Peptides more extensively acetylated in the strain over-expressing SCO0988 than in control strain.

Bright green: Peptides identified exclusively in the control strain.

Light Green: Peptides more extensively acetylated in control strain than in the strain over-expressing SCO0988.

“○” represent peptides detected at 24 h.

**“**●” represent peptides detected at 36 h.

Names in white of column A represent proteins detected at both 24 h and 36h.
